# Prognostic impact of sarcopenia on 5-year overall and progression-free survival in lung cancer patients: a prospective cohort study

**DOI:** 10.3389/fnut.2026.1727652

**Published:** 2026-02-04

**Authors:** Ting Zhao, Xin-qi Li, Zhan Shi, Chao-bao Zhang, Ying-gang Zhu

**Affiliations:** 1 Shanghai Key Laboratory of Clinical Geriatric Medicine, Department of Pulmonary and Critical Care Medicine, Shanghai Medical College, Shanghai Institute of Geriatrics and Gerontology, Huadong Hospital, Fudan University, Shanghai, China; 2 Department of Oncology, Huadong Hospital, Fudan University, Shanghai, China; 3 Shanghai Key Laboratory of Clinical Geriatric Medicine, Shanghai Medical College, Shanghai Institute of Geriatrics and Gerontology, Huadong Hospital, Fudan University, Shanghai, China; 4 National Clinical Research Center for Aging and Medicine, Huashan Hospital, Fudan University, Shanghai, China

**Keywords:** bioelectrical impedance, lung cancer, muscle function, muscle quality, sarcopenia

## Abstract

**Background:**

Sarcopenia is increasingly recognized as a critical prognostic factor in cancer patients, particularly in lung cancer, However, currently the relationship between Sarcopenia and lung cancer prognosis was primarily assessed using imaging modalities such as CT scans and its impact on outcomes in Chinese lung cancer patients, assessed using comprehensive Asian diagnostic criteria, remains underexplored. This study aimed to evaluate the association between Sarcopenia and tumor prognosis and outcome in lung cancer patients.

**Methods:**

A prospective cohort of 403 lung cancer patients admitted to Huadong Hospital (2020–2025) was analyzed. Sarcopenia was diagnosed using Asian Working Group for Sarcopenia (AWGS) criteria, combining muscle mass (bioelectrical impedance analysis), handgrip strength, and gait speed. Survival outcomes (overall survival [OS] and progression-free survival [PFS]) were compared between sarcopenic and non-sarcopenic groups using Kaplan–Meier and univariate and multivariate Cox regression analyses were used to identify independent predictors of OS and PFS.

**Results:**

Sarcopenia was identified in 43.2% of patients (174/403). Compared with non-sarcopenic patients, sarcopenic patients had significantly shorter median OS (13.2 vs. 43.3 months; *p* < 0.001) and PFS (11.5 vs. 25.4 months; *p* < 0.001). At baseline, sarcopenic patients were older (74.3 ± 7.7 vs. 71.0 ± 8.2 years, *p* < 0.001), had lower BMI (20.5 ± 2.9 vs. 23.5 ± 2.9 kg/m^2^, *p* < 0.001), poorer ECOG PS (1.4 ± 1.1 vs. 0.9 ± 0.8, *p* < 0.001), higher NRS-2002 (3.7 ± 1.6 vs. 2.6 ± 1.2, *p* < 0.001), lower handgrip strength (23.4 ± 7.1 vs. 30.8 ± 7.8 kg, *p* < 0.001), and slower walking speed (0.7 ± 0.3 vs. 1.0 ± 0.2 m/s, *p* < 0.001). In multivariable Cox regression, sarcopenia independently predicted worse OS (HR 2.33, 95% CI 1.64–3.33, *p* < 0.001) and PFS (HR 1.70, 95% CI 1.26–2.28, *p* < 0.001), with consistent trends across most subgroups. Only low BMI (OS *p* = 0.59; PFS *p* = 0.693), squamous cell carcinoma and other histology (OS *p* = 0.14; PFS *p* = 0.056), and I-II tumor stage (OS *p* = 0.098; PFS *p* = 0.682)showed no significant associations, while in patients with high body fat percentage the association with OS was not significant (*p* = 0.115) but remained significant for PFS (HR 1.68, 95% CI 1.03–2.72, *p* = 0.036).

**Conclusion:**

Sarcopenia is associated with reduced overall survival time and progression-free survival in lung cancer patients. Sarcopenia is an independent predictor of poor survival particularly in specific high-risk subgroups. When assessing for sarcopenia it is crucial to include assessment of muscle function in evaluating the prognosis of lung cancer.

## Introduction

Lung cancer currently ranks as the most prevalent malignancy and has the highest mortality among solid tumors (1). According to GLOBALCAN statistics, lung cancer accounted for the highest new cancer incidence in China in 2022, comprising approximately 40.8 cases per 100,000 population, and it was the primary cause of cancer mortality, with a death rate of about 26.7/100,000 (2). The main subtypes of lung cancer include non-small cell lung cancer (NSCLC) and small cell lung cancer (SCLC), with NSCLC representing 80–85% of all cases. Despite significant advancements in targeted and immunotherapies, the prognosis for advanced-stage lung cancer patients remains poor, with a 5-year survival rate of approximately 23% (1). Prognosis depends not only on tumor type, stage, and treatment response but also on the patient’s systemic condition.

Sarcopenia, an age-related progressive syndrome characterized by systemic loss of skeletal muscle mass and function (3), correlates with adverse health outcomes such as falls, fractures, functional decline, and increased mortality (4). As a manifestation of malnutrition and cachexia in cancer patients, Sarcopenia occurs more frequently in advanced NSCLC compared to other cancers (5). The prevalence of Sarcopenia across different cancer types ranges from 28.3 to 61% (6), with higher incidence in cancer populations than in community settings due to factors like reduced appetite, decreased physical activity, heightened metabolic demands from malignancy, therapeutic side effects, and cancer-related inflammation and oxidative stress that impair muscle regeneration (7). Numerous studies associate Sarcopenia with postoperative complications, increased chemotherapy toxicity, reduced treatment tolerability, and poor oncological outcomes (8, 9), significantly impacting quality of life and daily activity function.

Prior research using dual-energy X-ray absorptiometry (DXA) or bioelectrical impedance analysis (BIA) confirmed low muscle mass as an independent risk factor for mortality in adult cancer patients (10). CT imaging at the third lumbar vertebra (L3) to assess skeletal muscle index (L3-SMI) is commonly used for Sarcopenia diagnosis in lung cancer patients due to routine scan availability, though this method has limitations in fully evaluating muscle function (11, 12). Few studies have applied comprehensive Asian Sarcopenia diagnostic criteria, which include muscle mass, handgrip strength, and gait speed, to Chinese lung cancer populations. Thus, there is an urgent need for reliable, cost-effective screening tools to predict independent risk factor of adverse outcomes in lung cancer patients. This study aims to investigate the impact of Sarcopenia on survival prognosis in lung cancer patients and determine its role as an independent risk factor. Findings will inform risk stratification, guide personalized intervention strategies, and provide evidence-based insights for clinical practice.

## Methods

### Study design and patient selection

**Figure 1:**
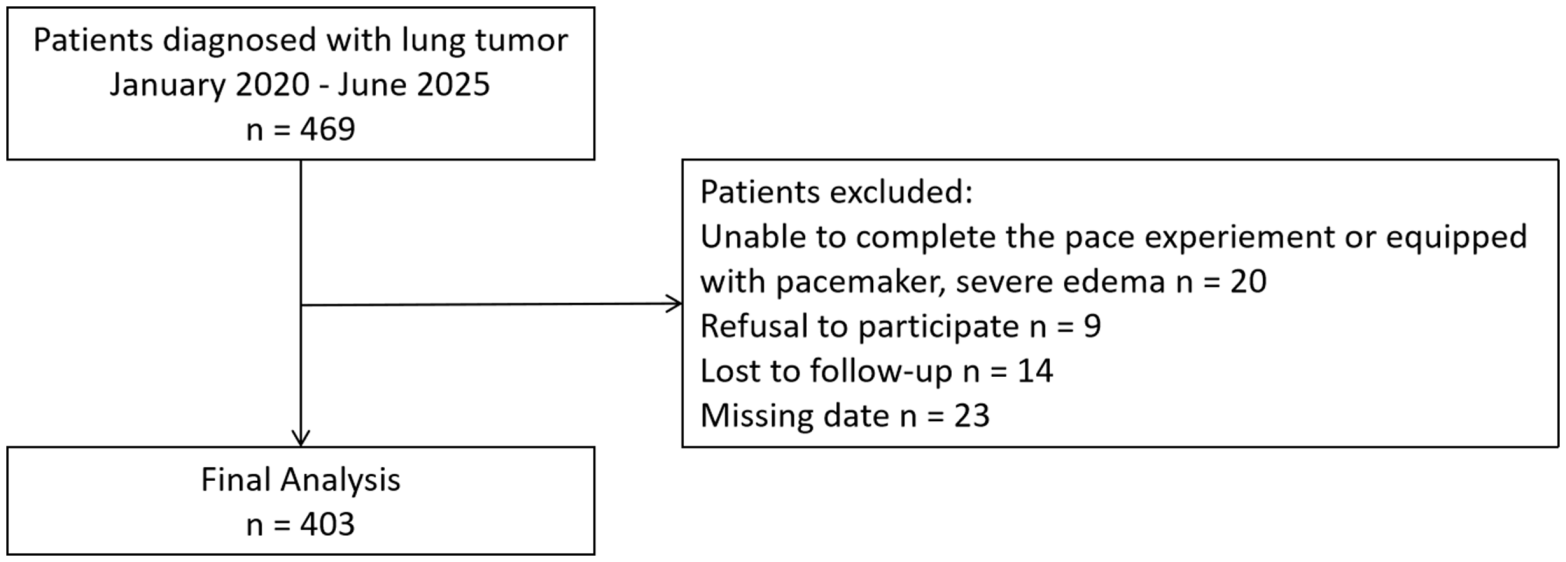
Enrollment flow diagram.

This prospective study enrolled consecutive adult patients with diagnosed lung tumors admitted to the Department of Respiratory Medicine at Huadong Hospital between January 2020 and April 2025. Patients were excluded if they were unable to complete the pace experiment, had a pacemaker, severe edema, refused to participate, or were lost to follow-up. After applying these exclusion criteria, a total of 403 inpatients were included in the final analysis, and the participant enrollment flowchart is presented in Figure 1. Participants meeting the predefined inclusion criteria were recruited after providing informed consent. The study complied with international ethical standards for human subject research and received approval from the Institutional Ethics Board of Huadong Hospital (ClinicalTrials.gov: NCT05212285). Follow-up continued through the study end date of April 2025.

### Data collection

Age, gender, oral medication use and comorbidities, history of smoking were obtained at admission. Tumor diagnosis was confirmed by consensus between two experienced physicians. Participants’ electronic medical records was used to obtain data on their systemic imaging for purposes of tumor staging (performed with the AJCC 8th edition), Histological classification, Molecular targeted gene results, Tumor markers and laboratory data cancer diagnosis, Eastern Cooperative Oncology Group (ECOG) performance status, and initial cancer treatment for analysis. The baseline comorbidity was assessed using age-adjusted Charlson Comorbidity Index (CCI) scores, Age-adjusted CCIs were calculated by adding one point for each decade over 40 years (13), nutritional status was assessed using the NRS 2002 (Nutritional Risk Screening 2002 scoring system).

### Body composition and assessment of sarcopenia

Diagnosis of Sarcopenia was based on the criteria of the Asian Working Group for Sarcopenia (14), Whole-body composition was assessed using bioelectrical impedance analysis (BIA; InBody 570, Korea). All measurements were performed in the morning after at least 8 h of fasting and before the initiation of intravenous fluid therapy. Patients were instructed to wear light clothing and to empty their bladders before the assessment. The BIA measurement provided data on body weight, body fat percentage, bone mineral content, and the skeletal muscle mass index (SMI). Body mass index (BMI) was calculated using the standard formula: BMI = body weight (kg)/[height (m)]^2^. BMI values were categorized into three groups based on international definitions for underweight, normal, and overweight, using 18.5 and 24.0 as cut-off points according to the revised Asia-Pacific BMI criteria by the World Health Organization (15). The cutoff of height-adjusted muscle mass <7.0 kg/m^2^ in men and <5.7 kg/m^2^ in women. Handgrip strength was measured utilizing a Jamar (USA) hydraulic hand dynamometer, one measurement was conducted for each arm, with participants seated in an upright position, maintaining a 90° elbow flexion. The average of the right and left values was derived and expressed in absolute units (kg). Low muscle strength is defined as handgrip strength <28 kg for men and <18 kg for women; Gait speed was used to assess physical performance, and low physical performance was defined as a 6-m walk speed <1.0 m/s, based on the criteria of the Asian Working Group for Sarcopenia (14).

### Follow-ups

Post-treatment follow-up with a history and physical examination along with systemic imaging (contrast-enhanced CT, PET, and/or MRI) was performed every 6 weeks for the first year, every 3 months for the following 5 years, and every 6 months thereafter. A minimum follow-up duration of 3 months was required for inclusion in the final analysis. Lost to follow-up was defined as the inability to obtain outcome information at the planned 3-month follow-up time point, despite the absence of relevant clinical data in the electronic medical record and unsuccessful contact after repeated telephone attempts. OS (Overall survival) was defined as the time from diagnosis to the date of death by any cause. PFS (Progression-Free Survival) was defined as the time from diagnosis to the date of confirmed disease progression (defined by RECIST criteria) or death, whichever occurred first.

### Sample size and sensitivity analysis

The *a priori* sample size for overall survival was estimated using Schoenfeld’s method, assuming a hazard ratio of 1.5, a sarcopenia prevalence of 40%, and a 5-year event rate of 75%. This yielded a required sample size of approximately 265 patients; with 403 patients included, the study was adequately powered.

### Statistical analysis

For continuous variables, standard deviations (SDs) or medians with interquartile range (Q1, Q3) for descriptive purposes, if the data exhibited a normal distribution, they were analyzed using the unpaired Student’s *t*-test. If the data, as determined by the Shapiro–Wilk test, did not exhibit a normal distribution, the Mann–Whitney U test was employed as the nonparametric alternative. The OS and PFS between different groups was compared by Kaplan–Meier analysis with the log-rank test. Univariate and multivariate Cox regression analyses were implemented to identify independent predictors of OS and PFS. Any variable where *p* < 0.1 in univariable analysis was considered a candidate for multivariable analysis. Hazard ratios and 95% confidence intervals (CI) were generated.

To assess potential heterogeneity of the association between sarcopenia and survival outcomes, interaction terms between sarcopenia and selected clinical variables (age stratification, sex, histological type, tumor stage, and chemotherapy status) were incorporated into multivariable Cox proportional hazards models. Selected clinical variables were chosen based on clinical relevance and data availability. *p* values for interaction were calculated using the Wald test.

## Result

From January 2020 to April 2025, a total of 469 patients who diagnosed with lung cancer patients were included in our study, The flowchart is shown in Figure 1. The median follow-up duration for OS and PFS was 24.1 months (95%CI 19.1–29 months) and 18.1 months (95%CI 14.2–22 months), respectively. Table 1 delineates the clinical characteristics of the patients. The median age of the participants was 72.2 ± 8.2 years, ranging from 42 to 94 years. Male patients accounted for 71.1% of the cohort. The mean BMI was 22.2 ± 3.2 kg/m^2^. Current smokers accounted for 43 cases (10.7%). Histologically, 65% had adenocarcinoma, 24.8% squamous cell carcinoma, and 8.4% small cell lung cancer, while 1.7% included other types (e.g., large cell carcinoma and sarcomatoid carcinoma). By disease stage, 20.8% were stage I-II and 79.2% stage III-IV. The mean ECOG-PS score was 1.2 ± 1.0 (see Table 1).

**Table 1:** Baseline demographic and clinical characteristic of patients with and without sarcopenia.

Characteristics	All	Sarcopenia	Non-Sarcopenia	*p*-value
	*N* = 403	*N* = 174	*N* = 229	
Age, years, mean (SD)	72.2 (8.2)	74.3 (7.7)	71 (8.2)	<0.001
Sex				0.07
Male *n* (%)	289 (71.7)	133 (76.4)	156 (68.1)	
Female *n* (%)	114 (28.3)	41 (23.6)	73 (31.9)	
BMI, kg/m^2^, mean (SD)	22.2 (3.2)	20.5 (2.9)	23.5 (2.9)	<0.001
SMI, kg/m^2^, median (p.25, 75)	6.8 (6.1,7.4)	6.4 (5.8,6.9)	7.3 (6.5,7.7)	<0.001
Handgrip, kg, mean (SD)	27.4 (8.4)	23.4 (7.1)	30.8 (7.8)	<0.001
Normal *n* (%)	260 (64.5)	60 (23.1)	200 (76.9)	
Decreased *n* (%)	143 (35.5)	114 (79.7)	29 (20.3)	
Walking speed, m/s, mean (SD)	0.9 (0.3)	0.7 (0.3)	1.0 (0.2)	<0.001
≤0.8 *n* (%)	161 (40)	117 (67.2)	44 (19.2)	
>0.8 *n* (%)	242 (60)	57 (32.8)	185 (80.8)	
Smoking status *n* (%)				0.047
Smoker	43 (10.7)	11 (6.3)	32 (14)	
Ex-smoker	134 (33.3)	60 (34.5)	74 (32.3)	
Non-smoker	226 (56.1)	103 (59.2)	123 (53.7)	
Quantity of oral medicine, mean (SD)	2.5 (2.1)	2.7 (2.2)	2.4 (1.9)	0.094
Hypertension *n* (%)	197 (48.9)	78 (44.8)	119 (52)	0.156
Diabetes mellitus *n* (%)	82 (20.3)	33 (19)	49 (21.4)	0.361
aCCI, mean (SD)	7.8 (2.4)	8.3 (2.3)	7.5 (2.4)	0.038
Body fat percentage,%, mean (SD)	24.2 (8.2)	22.6 (8.4)	25.3 (7.8)	0.001
Bone mineral content, g, mean (SD)	3.1 (0.5)	2.9 (0.4)	3.2 (0.5)	<0.001
Other solid tumors *n* (%)	31 (7.7)	10 (5.7)	21 (9.2)	0.258
NRS2002 Score, mean (SD)	3.1 (1.5)	3.7 (1.6)	2.6 (1.2)	<0.001
Historical type *n* (%)				0.146
Adenocarcinoma	262 (65)	113 (64.9)	149 (65.1)	
Squamous cell carcinoma	100 (24.8)	49 (28.2)	51 (22.3)	
Small cell lung cancer	34 (8.4)	11 (6.3)	23 (10.0)	
Others including Sarcomatoid carcinoma	7 (1.7)	1 (0.6)	6 (2.6)	
TNM stage *n* (%)				
T1	76 (18.9)	24 (13.8)	52 (22.7)	0.082
2	100 (24.8)	43 (24.7)	57 (24.9)	
3	93 (23.1)	40 (23)	53 (23.1)	
4	134 (33.3)	67 (38.5)	67 (29.3)	
N0	134 (33.3)	46 (26.4)	88 (38.4)	0.037
1	47 (11.7)	18 (10.3)	29 (12.7)	
2	127 (31.5)	64 (36.8)	63 (27.3)	
3	95 (23.6)	46 (26.4)	49 (21.4)	
M0	158 (39.2)	52 (29.9)	106 (46.3)	0.001
1	245 (60.8)	122 (70.1)	123 (53.7)	
Cancer stage *n* (%)				0.048
I-II	84 (20.8)	28 (16.1)	56 (24.5)	
III-IV	319 (79.2)	146 (83.9)	173 (75.5)	
ECOG PS, mean (SD)	1.2 (1.0)	1.4 (1.1)	0.9 (0.8)	<0.001
Treatment *n* (%)				
Molecular targeted	176 (43.7)	79 (45.4)	97 (42.4)	0.545
Immunotherapy	178 (44.2)	83 (47.7)	95 (41.5)	0.225
Chemotherapy	218 (54.1)	82 (47.1)	136 (59.4)	0.016
Radiation therapy	64 (15.9)	29 (16.7)	35 (15.3)	0.783
Molecular targeting *n* (%)				
*EGFR*	122 (58.3)	46 (44.7)	76 (57.6)	0.065
*Alk*	1 (0.2)	0	1 (0.9)	0.362
*Ros1*	2 (0.5)	0	2 (3.1)	0.180
*Kras*	20 (5)	6 (66.7)	14 (60.9)	0.761
*Braf*	3 (0.7)	2 (11.8)	1 (2.9)	0.255
Tumor Marker mean (SD)				
CEA, ng/ml	49 (178.8)	48.2 (182.2)	50.1 (174.4)	0.92
Cyfra211, ng/ml	9.9 (23.8)	7.4 (13.4)	13.4 (32.9)	0.017
SCC, ng/ml	2.7 (6.8)	2.2 (4.3)	3.4 (8.9)	0.137
NSE, ng/ml	20.1 (25.9)	19.8 (21.9)	21.1 (30.5)	0.657
ALB, g/L, mean (SD)	39.2 (5.5)	37.8 (5.3)	40.3 (5.4)	<0.001

Data are expressed as mean (standard deviations), *n* (%) or median (interquartile range Q1, Q3); BMI, body mass index; SMI, skeletal muscle mass index; aCCI, age adjusted Charlson Comorbidity Index; NRS2002 Score, Nutritional Risk Screening 2002 scoring system; TNM stage, tumor-node-metastasis stage; ECOG PS, ECOG performance Status; CEA, carcinoembryonic antigen; Cyfra211, Cytokeratin 19 Fragment; SCC, Squamous Cell Carcinoma Antigen; NSE, Neuron-Specific Enolase; ALB, Albumin.

A total of 174 (43.2%) individuals were diagnosed with Sarcopenia in lung tumor patients, the baseline demographic and Clinical Characteristics comparison between patients with and without Sarcopenia were summarized in Table 1. Compared with non-sarcopenic group, those who were diagnosed with Sarcopenia were older (74.3 years vs. 71 years, *p* < 0.001), had a lower mean BMI value (20.5 kg/m^2^ vs. 23.5 kg/m^2^, *p* < 0.001), percentage of body fat (22.6% vs. 25.3%, *p* = 0.001) and bone mineral content (2.9 g vs. 3.2 g, *p* < 0.001). As for muscle mass and muscle function, in patients with Sarcopenia, the SMI was lower than those without Sarcopenia (6.4, IQR 5.8–6.9 vs. 7.3, IQR 6.5–7.7 kg/m^2^), respectively, *p* < 0.01, significant differences were observed in the Sarcopenia group, with lower mean handgrip strength (23.4 ± 7.1 kg vs. 30.8 ± 7.8 kg) and reduced gait speed (0.7 ± 0.3 m/s vs. 1.0 ± 0.2 m/s) compared to non-sarcopenic group (all *p* < 0.001). In the sarcopenic group, the proportion of patients treated with chemotherapy was significantly higher (47.1% vs. 59.4%) than non-sarcopenic group, *p* = 0.016, Patients with Sarcopenia demonstrated significantly higher rates of tumor metastasis (70.1% vs. 53.7%), poorer ECOG PS scores (1.4 ± 1.1 vs. 0.9 ± 0.8, *p* < 0.001), elevated NRS 2002 scores (3.7 ± 1.6 vs. 2.6 ± 1.2, *p* < 0.001), and higher CCI score (8.3 ± 2.3 vs. 7.5 ± 2.4). Laboratory analyses revealed significantly lower levels of serum Cyfra 21-1 and albumin in the sarcopenic group compared to non-sarcopenic patients. No statistically significant differences were observed between the two groups of other treatment modalities, the proportion of diabetes mellitus and hypertension, tumor marker levels (CEA, NSE, SCC), and molecular testing results (*p* > 0.05).

**Figure 2:**
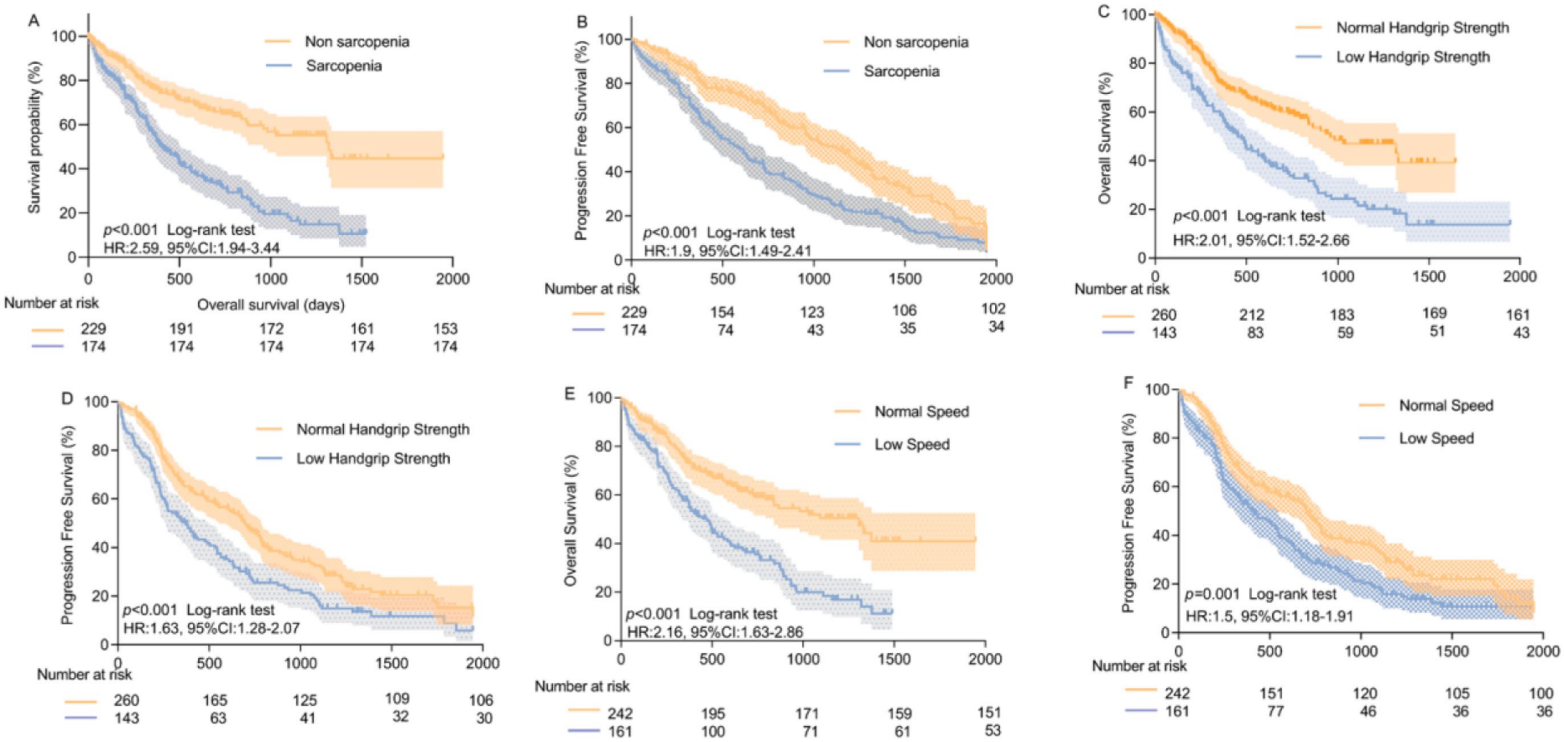
Analyses of OS and PFS among lung cancer patients with different muscle function and with or without sarcopenia. **(A)** Kaplan–Meier analyses of OS among lung cancer patients with and without sarcopenia; **(B)** Kaplan–Meier analyses of PFS among patients with and without sarcopenia; **(C)** Kaplan–Meier analyses of OS among patients with normal and low handgrip strength; **(D)** Kaplan–Meier analyses of PFS OS among patients with normal and low handgrip strength; **(E)** Kaplan–Meier analyses of OS among patients with normal and low gait speed; **(F)** Kaplan–Meier analyses of PFS among patients with normal and low gait speed. OS, overall survival; PFS, progression-free survival.

The median follow-up lasted 24.7 months (95% CI: 23.2–26.2 months), 199 deaths (49.4%) had occurred in the study population at the time of last follow up, the 1-year, 3-year, and 5-year overall survival (OS) rate were 66.9, 50.2, and 27.2%, respectively. The median OS of sarcopenic patients was 13.2 months (95% CI: 10.0–16.4) and significantly shorter compared with 43.3 months (95% CI: 32.2–54.4) in non-sarcopenic group (log-rank *p* < 0.001, Figure 2A), The 1-year, 3-year, and 5-year Progression free survival (PFS) rate were 60.1, 39.8, and 9.5%, respectively. The median PFS of sarcopenic patients were 11.5 months (95% CI: 8.5–14.5) and significantly shorter compared with 25.4 months (95% CI: 22.4–28.4) in non-sarcopenic group (log-rank *p* < 0.001, Figure 2B). When patients were stratified based on handgrip strength (using Sarcopenia-defined cutoffs), compared with normal handgrip, lower levels of handgrip were associated with a worse overall survival and tumor progression, with median OS of 14.9 vs.31.5 months (*p* < 0.001), median PFS of 12.2 vs. 23 months (*p* < 0.001) (Figures 2C,D). Compared with normal gait speed (>0.8 m/s), lower gait speed (<0.8 m/s) was associated with worse overall survival and progression-free survival, with a median OS of 15.6 vs. 43 months (*p* < 0.001) and a median PFS of 13.6 vs. 23.2 months (*p* < 0.001). In addition, handgrip strength and gait speed were both identified as independent prognostic factors for overall survival in the multivariable Cox regression analysis (as shown in Figures 2E,F).

Predicting variables, including age, sex, smoking status, quantity of oral medication, hypertension, aCCI, body composition (BMI, body fat percentage), Sarcopenia, tumor characteristics [staging, ECOG PS, tumor markers, nutritional status (NRS2002 score and ALB levels)] were examined by univariable analysis for OS and PFS. Univariable analysis revealed that Sarcopenia (*p* < 0.01), BMI < 18.5 kg/m^2^, quantity of oral medication, CCI score, body fat percentage, NRS2002 score, ECOG PS, CEA, Cyfra211, SCC, NSE, ALB levels were independently associated with OS and PFS (all *p* < 0.05), besides, age was also associated with overall survival. After adjustment for the aforementioned confounding factors, Sarcopenia still proved to be an independent prognostic factor of OS in the multivariate Cox regression analysis (HR 2.33, 95%CI 1.64–3.33, *p* < 0.001) and PFS (HR 1.7, 95%CI 1.26–2.28, *p* < 0.001), aCCI, ECOG PS and NSE level was significant independent predictor of OS among patients (Table 2). aCCI, cancer stage, NSE level and ALB level was significant independent predictor of PFS.

**Table 2:** Univariable and multivariable Cox hazards regression analysis for overall survival and progression free survival.

Variable	Ref.	OS				PFS			
		Univariable	*p*-value	Multivariable	*p*-value	Univariable	*p*-value	Multivariable	*p*-value
		HR (95% CI)		HR (95% CI)		HR (95% CI)		HR (95% CI)	
Sarcopenia,	None	2.59 (1.94–3.44)	<**0.001**	2.33 (1.64–3.33)	<**0.001**	1.9 (1.49–2.41)	<**0.001**	1.7 (1.26–2.28)	<**0.001**
Age		1.03 (1.01–1.05)	**0.002**			1.01 (0.99–1.02)	0.403		
Sex	Male	1.31 (0.96–1.8)	0.094			0.87 (0.66–1.13)	0.290		
Smoking status	None smoker								
Smoker		1.25 (0.93–1.67)	0.64			0.86 (0.55–1.32)	0.478		
Ex-smoker		0.88 (0.51–1.51)	0.138			1.07 (0.82–1.38)	0.621		
BMI	18.5–24.9	0.93 (0.89–0.98)	**0.002**						
<18.5		1.75 (1.18–2.6)	**0.006**			1.53 (1.08–2.17)	**0.017**		
>24.9		0.74 (0.53–1.03)	0.075			0.76 (0.57–1.00)	0.052		
Quantity of oral medicine		1.07 (1.01–1.14)	**0.033**			1.08 (1.02–1.14)	**0.014**		
Hypertension	None	1.01 (0.76–1.33)	0.96			0.86 (0.68–1.1)	0.22		
aCCI		1.25 (1.17–1.33)	<**0.001**	1.21 (1.11–1.32)	<**0.001**	1.19 (1.13–1.25)	<**0.001**	1.1 (1.03–1.20)	**0.008**
BFP		0.98 (0.96–0.99)	**0.013**			0.98 (0.97–1.00)	**0.029**		
Other solid tumors	None	0.87 (0.50–1.49)	0.605			1.05 (0.68–1.63)	0.83		
NRS2002 Score		1.16 (1.06–1.28)	**0.001**			1.1 (1.01–1.19)	**0.036**		
Cancer stage III-IV	I-II	3.1 (1.95–4.88)	<**0.001**			2.28 (1.94–3.97)	<**0.001**	1.11 (1.03–1.2)	**0.008**
ECOG PS		1.51 (1.33–1.72)	<**0.001**	1.33 (1.12–1.59)	<**0.001**	1.44 (1.28–1.61)	<**0.001**		
CEA		1.00 (1.00–1.001)	<**0.001**			1.0 (1.0–1.001)	**0.002**		
Cyfra211		1.01 (1.00–1.01)	<**0.001**			1.01 (1.005–1.01)	<**0.001**		
SCC		1.02 (1.01–1.04)	**0.009**			1.02 (1.01–1.04)	**0.012**		
NSE		1.02 (1.01–1.02)	<**0.001**	1.01 (1.01–1.02)	**<0.001**	1.01 (1.01–1.02)	<**0.001**	1.01 (1.01–1.02)	<**0.001**
ALB		0.93 (0.91–0.95)	**<0.001**			0.95 (0.93–0.97)	<**0.001**	0.97 (0.93–1.0)	**0.026**

HR, Hazard Ratio; BMI, body mass index; aCCI, age adjusted Charlson Comorbidity Index; BFP, body fat percentage; NRS2002 Score, Nutritional Risk Screening 2002 scoring system; ECOG PS, ECOG performance Status; CEA, carcinoembryonic antigen; Cyfra211, Cytokeratin 19 Fragment; SCC, Squamous Cell Carcinoma Antigen; NSE, Neuron-Specific Enolase; ALB, Albumin. Bold values indicate statistical significance at the *p* < 0.05 level.

### Subgroup analysis

**Figure 3:**
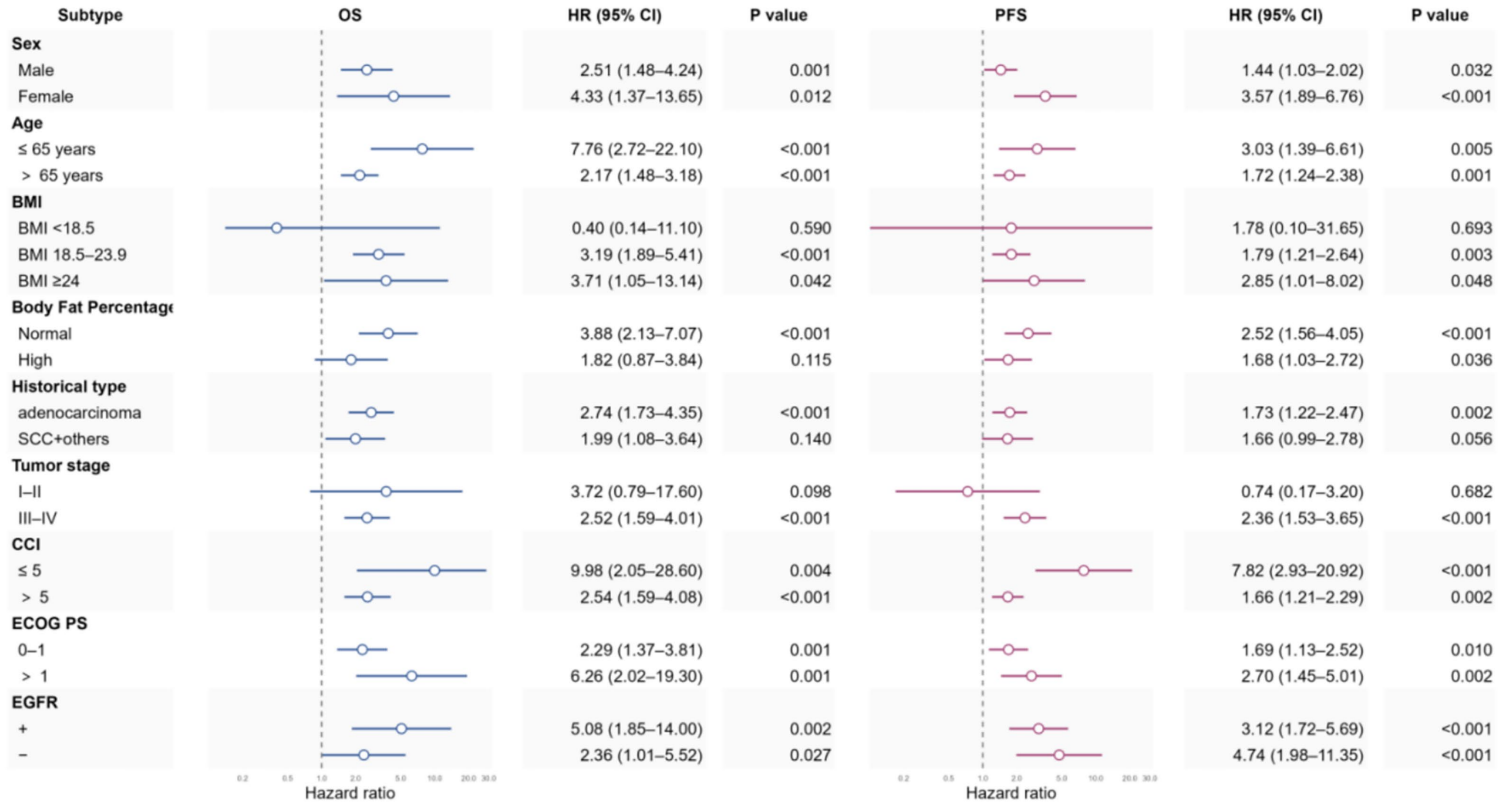
Forest plots illustrating stratified analyses of association of sarcopenia and OS/PFS. OS, overall survival; PFS, progression-free survival; ECOG-PS, Eastern Cooperative Oncology Group performance status; CCI, Charlson Comorbidity Index; SCC + others, squamous cell carcinoma (SCC) and other histologies.

The association between sarcopenia and OS/PFS was evaluated across prespecified subgroups including sex, age, BMI, body fat percentage (BFP), tumor histology and stage, EGFR status, aCCI, and ECOG PS (Figure 3). Due to the small number of small cell lung cancer and other rare subtypes, these were grouped with squamous cell carcinoma in multivariable subgroup analyses.

In summary, sarcopenia was associated with worse OS and PFS in most subgroups. Exceptions included low BMI (<18.5 kg/m^2^) and stage I–II, where neither OS nor PFS associations were statistically significant, and squamous cell carcinoma/other histologies, where the OS association was not significant and the PFS association was borderline (*p* = 0.056). Notably, high body fat percentage attenuated the OS association (non-significant) while the PFS association remained significant. Besides normal Body fat percentage categories and III-IV tumor stage categories, in which Sarcopenia showed highly statistically significant associations with both OS and PFS (*p* < 0.001), the detrimental impact of sarcopenia on OS and PFS appeared more pronounced in subgroups such as females, younger patients (≤65 years), those with BMI ≥ 18.5 kg/m^2^, normal body fat percentage, adenocarcinoma histology, advanced stage (III–IV), aCCI ≤ 5, PS > 1, and EGFR-positive status. Other subgroup details are provided in Figure 3 for reference.

### Effect modification analyses

Significant effect modification was observed between sarcopenia and several clinical variables, including age, sex, histological type, tumor stage, and chemotherapy status, for both OS and PFS (all P for interaction <0.05; Table 3).

**Table 3:** Interaction analyses between sarcopenia and clinical variables for overall survival and progression-free survival based on Cox proportional hazards models.

Interaction term	OS	PFS		
	HR (95%CI)	*P* values for Interaction	HR (95%CI)	*P* values for Interaction
Sarcopenia*age stratification	2.10 (1.38–3.21)	0.001	1.72 (1.18–2.51)	0.005
Sarcopenia*sex stratification	2.27 (1.52–3.36)	<0.001	1.43 (1.03–2.0)	0.034
Sarcopenia*historical type	2.63 (1.67–4.15)	<0.001	2.17 (1.46–3.23)	<0.001
Sarcopenia*tumor stage	2.67 (1.65–4.32)	<0.001	2.05 (1.36–3.9)	0.001
Sarcopenia*chemo therapy	1.80 (1.14–2.86)	0.012	1.70 (1.12–2.58)	0.014

Hazard ratios represent the effect of sarcopenia within each stratified variable, while *P* values for interaction were derived from models including the corresponding interaction term. Age stratification: ≤65 years vs. >65 years; Sex stratification: male vs. female; Historical type: Non-small cell lung cancer vs. small cell lung cancer; Tumor stage: stage I-II vs. stage III-IV.

## Discussion

Our findings indicate that the prevalence of Sarcopenia is 43.2%, slightly lower than previously reported in the literature (16). Yang et al.’s systematic review and meta-analysis (17) pooled data from 13 studies involving a total of 1,810 patients, concluding a combined prevalence of 45% (95% CI: 32–57%). Prior reviews and meta-analyses have demonstrated that Sarcopenia is an unfavorable prognostic factor associated with overall survival (OS) in patients with small cell lung cancer (SCLC) or various stages of non-small cell lung cancer (NSCLC). However, only two studies have reported on progression-free survival (PFS) (18, 19). Another key aspect is that our study reinforces Sarcopenia as an independent prognostic factor for both OS (HR = 2.33, 95% CI: 1.64–3.33) and PFS (HR = 1.7, 95% CI: 1.26–2.28). Although several studies have investigated the relationship between lung cancer prognosis and skeletal muscle mass, few have simultaneously assessed muscle mass and strength. Previous research involving surgical patients and NSCLC cases primarily evaluated muscle mass alone (17, 20, 21). While CT-based estimation of muscle mass is valuable and suitable for clinical settings, since CT imaging is a routine, minimally invasive method for tumor assessment, accurate measurement of muscle mass still requires trained personnel or specialized software, which can be costly and less reproducible. Importantly, these assessments do not incorporate muscle function or strength, which are critical for a comprehensive diagnosis of Sarcopenia, it is insufficient for accurately diagnosing Sarcopenia. Patients diagnosed solely based on imaging-defined Sarcopenia (radiologically defined Sarcopenia) (22) only reflect muscle quantity, as muscle strength correlates only moderately with cross-sectional area and muscle thickness (23), combining reduced muscle strength and mass provides a more comprehensive reflection of the true status of Sarcopenia (24). Our study combines assessments of grip strength and gait speed—two vital elements for Sarcopenia diagnosis—along with whole-body bioelectrical impedance analysis (BIA) to measure body fat percentage and total muscle mass, providing a more comprehensive evaluation. Although comprehensive sarcopenia assessment is ideal, even partial assessments (e.g., grip strength or gait speed alone) are feasible in routine clinical practice, including in frail or advanced-stage patients, and still provide valuable prognostic information. To our knowledge, few reports have addressed the prognosis of complete Sarcopenia in lung cancer populations. Our research demonstrates that Kaplan–Meier curve analysis reveals grip strength and gait speed have a significant impact on patients’ overall survival (OS) and progression-free survival (PFS) (log-rank *p* < 0.01), emphasizing the critical prognostic role of muscle strength and gait speed, suggesting that interventions such as nutritional support, exercise, or other modalities, are necessary to improve patient outcomes. Given the critical prognostic significance of grip strength and gait speed, future investigations should explore intervention strategies to validate these approaches.

The finding that Sarcopenia is significantly associated with overall survival aligns with previous research (25–27). Advanced cancer patients often face declining functional capacity, pain, malnutrition, and cachexia. Our research demonstrates that hypoproteinemia is associated with shorter progression-free survival (PFS), while performance status (PS) scores correlate with overall survival (OS) in lung cancer. PS, as an independent prognostic factor, may be influenced by subjective assessment (28). When applying comprehensive diagnostic criteria for Sarcopenia, including muscle strength, a significant association with poor survival outcomes in lung cancer patients was observed. This association remained independent after adjustment for nutritional scores, physical performance, and tumor stage. These findings suggest that patients may experience detrimental muscle loss and functional decline even when nutritional status or comorbidities are not severe. Therefore, assessment of sarcopenia may partially compensate for the limitations of subjective clinical evaluations. Future efforts could involve integrating several key indicators to develop more accurate prognostic models for lung cancer, enabling better assessment to stratify risk and guide personalized therapy.

In stratified analyses, heterogeneity was observed in the association between sarcopenia and prognosis across age, sex, histological type, tumor stage, and chemotherapy status. While previous studies have emphasized the importance of screening for sarcopenia prior to cancer treatment, particularly in patients with limited therapeutic options (29), the biological mechanisms underlying cancer-associated sarcopenia are complex and may involve systemic inflammation and accelerated muscle proteolysis (30). Importantly, these subgroup findings should be interpreted as exploratory and indicative of potential heterogeneity rather than definitive evidence of stronger prognostic effects in specific populations. Consistent with this rationale, interaction analyses with tumor stage and chemotherapy status were conducted to aid interpretation of heterogeneity rather than to derive fully stratified or interaction-driven prognostic models, given concerns regarding event numbers and model stability. The primary objective of the present study was not to compare the magnitude of sarcopenia-related risk across subgroups, but to assess its overall prognostic relevance in lung cancer.

Previous studies have shown that the prognostic significance of Sarcopenia varies between resectable and unresectable lung cancers. Meta-analyses of resectable cases have demonstrated a significant association between Sarcopenia and overall survival (31, 32), whereas in unresectable stage IIIB/IV NSCLC, Sarcopenia’s correlation with SMI diminishes, likely due to disease stage effects. Our stratified analysis suggests that the prognostic relevance of sarcopenia may differ across disease stages, with more pronounced associations observed in stage III–IV disease. The discrepancy may be due to differences in population characteristics and covariates included. Furthermore, our study incorporates assessments of muscle function alongside SMI, which may enhance prognostic accuracy in advanced-stage patients. Patients with late-stage disease often require more aggressive treatments, such as combined chemotherapy and immunotherapy. Sarcopenic patients, due to limited metabolic reserves and decreased drug clearance, are more susceptible to dose-limiting toxicities like myelosuppression and hepatotoxicity, leading to treatment interruptions or reduced efficacy. Early-stage patients typically receive less intensive therapy, such as monotherapy or surgery, which may obscure the impact of Sarcopenia. Although preoperative CT scans routinely evaluate tumor staging with minimal additional effort, incorporating simple physical assessments like grip strength and gait speed alongside muscle mass evaluation could be particularly valuable for late-stage and poor PS patients. These findings further support the relevance of incorporating functional muscle assessments into prognostic evaluation, particularly in patients with advanced disease or poor performance status. These results imply that Sarcopenia may serve as a “hidden” determinant of treatment response, even in less aggressive disease presentations. The interaction between Sarcopenia and tumor biology, including cachexia pathways and systemic inflammation, warrants further investigation to elucidate mechanisms linking muscle loss to tumor progression.

In exploratory analyses, we observed evidence suggesting that histological subtype may modify the prognostic impact of sarcopenia. However, this study was not specifically designed or powered to evaluate histology-specific effects, and the interaction analysis should therefore be interpreted with caution. Future studies with dedicated designs are warranted to further clarify whether the prognostic relevance of sarcopenia differs between NSCLC and SCLC. Given the limited sample size in molecularly defined subgroups, these findings should be interpreted cautiously. In descriptive subgroup analyses, differential outcome patterns were observed between EGFR + and EGFR—patients, although formal interaction testing was not performed. Cancer-associated sarcopenia has been linked in prior studies to dysregulation of the AKT/mTOR signaling pathway, a central regulator of cellular growth, metabolism, and survival (33, 34). Potential biological crosstalk between EGFR signaling and AKT/mTOR–related metabolic pathways has been proposed in the literature, and may represent a mechanistic hypothesis warranting further investigation in lung cancer–associated sarcopenia.

This study has several limitations. First, although the AWGS guidelines recommend using bioelectrical impedance analysis (BIA) to measure skeletal muscle mass—a safe and non-invasive method for assessing whole-body composition—its accuracy remains slightly inferior to computed tomography (CT) or dual-energy X-ray absorptiometry (DXA). We excluded patients with severe edema and standardized measurements in the early morning to minimize confounding factors. We also note that BIA measurements can be affected by hydration status and systemic inflammation, which may influence accuracy in cancer patients. Second, in cancer patients, whether the muscle mass threshold measured by BIA is lower than in the general population warrants further investigation. Although sarcopenia was defined using the AWGS criteria in this study, emerging evidence suggests that disease- or stage-specific cutoff values for skeletal muscle mass may further improve prognostic stratification in lung cancer (35). This warrants investigation in future studies specifically designed for cutoff derivation and validation. Furthermore, our study population was derived from hospitalized patients at a single medical center, which may induce admission rate bias, and the applicability of the findings to community-based cancer patients remains uncertain. Third, sarcopenia and body composition were assessed exclusively at baseline, at the time of initial lung cancer diagnosis and prior to the initiation of any anticancer treatment. This baseline assessment reflects patients’ underlying physiological reserve and vulnerability, providing prognostic information independent of subsequent treatment effects, which is a strength of the current design. However, longitudinal reassessment of sarcopenia was not performed due to practical constraints, including limited resources and follow-up feasibility; key survival events were instead captured through predefined time points and simple clinical or telephone follow-up. As a result, we are unable to distinguish whether early changes in muscle parameters during follow-up would represent true sarcopenia progression, treatment-related effects (e.g., chemotherapy-induced toxicity), or transient physiological fluctuations. Future studies incorporating repeated assessments at clinically meaningful intervals (e.g., every 3–6 months) are warranted to characterize dynamic changes in sarcopenia, differentiate treatment-related effects from disease progression, and identify optimal follow-up time points for prognostic evaluation. Fourth, lifestyle factors, particularly dietary intake and nutrition, could not be fully evaluated, and these variables may confound the development and progression of Sarcopenia. Additionally, sarcopenia in our cohort likely reflects a combination of age-related (primary) and cancer-associated (secondary) muscle loss. While age was adjusted for in multivariable models, statistical correction alone cannot fully separate these processes, which may synergistically exacerbate muscle wasting. Future studies incorporating age-matched healthy controls or longitudinal assessments are warranted to better delineate the relative contributions of primary and secondary sarcopenia.

In conclusion, this study demonstrated that Sarcopenia was an independent prognostic factor associated with reduced overall survival and progression-free survival in patients with lung cancer. Subgroup trends were observed in age, sex, histological type, tumor stage, and chemotherapy status; these findings should be interpreted as exploratory given the limited sample sizes. Formal interaction analyses were conducted only for these variables, and other subgroups, including molecularly defined subgroups such as EGFR+, were not analyzed. Decreased skeletal muscle mass and diminished muscle strength serve as significant predictors of adverse clinical outcomes. This finding allows for early risk stratification of high-risk cohorts, which in turn facilitates timely intervention and may improve prognostic outcomes.

## Data Availability

The original contributions presented in the study are included in the article/supplementary material, further inquiries can be directed to the corresponding authors.
